# Recent advances in membrane vesicles of *Streptococcus mutans*: biogenesis, regulations, and functions

**DOI:** 10.1080/20002297.2026.2735102

**Published:** 2026-09-26

**Authors:** Han Wang, Yuqing Chen, Huilin Huang, Ruonan Chen, Shuai Chen, Xiaojing Huang

**Affiliations:** a Fujian Key Laboratory of Oral Diseases & Fujian Provincial Engineering Research Center of Oral Biomaterial & Stomatological Key Lab of Fujian College and University, School and Hospital of Stomatology, Fujian Medical University, Fuzhou, People's Republic of China; b Research Center of Oral Tissue Engineering & Institute of Oral Homeostasis, School and Hospital of Stomatology, Fujian Medical University, Fuzhou, People's Republic of China

**Keywords:** *Streptococcus mutans*, membrane vesicles, biogenesis, compositional heterogeneity, biological functions

## Abstract

**Background:**

*Streptococcus mutans* (*S. mutans*) is a major cariogenic pathogen in the oral cavity that actively releases membrane vesicles (MVs) encapsulating proteins, lipids, and nucleic acids. These highly heterogeneous MVs not only help regulate homeostasis of the local oral microecology but may also affect systemic health. A comprehensive review of the physiological characteristics of *S. mutans* MVs is therefore of considerable research value.

**Objective:**

By summarizing the latest advances in research on *S. mutans* MVs, this review provides a broad overview of the relevant studies to elucidate their physiological and pathological characteristics.

**Design:**

We conducted an extensive literature search in PubMed, Web of Science, and Google Scholar. The search strategy combined terms related to MVs and *S. mutans*, including ‘membrane vesicles,’ ‘extracellular vesicles,’ ‘bacterial extracellular vesicles,’ ‘microbial extracellular vesicles,’ ‘MVs,’ ‘EVs,’ ‘bEVs,’ ‘MEVs,’ ‘*Streptococcus mutans*,’ ‘*S. mutans*,’ ‘Gram-positive,’ ‘biogenesis,’ ‘cargo,’ and ‘biological functions.’ Literature published from database inception to August 2026 was included. This review delineates the biogenesis, cargo composition, and functions of MVs, and highlights current research challenges and future directions.

**Results:**

*S. mutans* MV biogenesis is driven jointly by environmental signals and bacterial gene regulatory networks. As efficient delivery vehicles for toxins and various bioactive factors, MVs constitute a core molecular tool through which *S. mutans* adapts to the complex oral microenvironment, competes with other species, and initiates pathogenic processes. MVs not only regulate the balance of the local oral microecology but also serve as key mediators of intermicrobial signaling and microbe–host cross-kingdom interactions. Deciphering the pathophysiological mechanisms of *S. mutans* MVs may revolutionize caries prevention and control strategies, providing a theoretical basis for developing novel anticaries vaccines and oral targeted therapies, while also offering new research entry points for elucidating the intrinsic links between oral infections and systemic diseases.

**Conclusions:**

*S. mutans* MV biogenesis is driven jointly by environmental signals and bacterial gene regulatory networks. As efficient delivery vehicles for toxins and various bioactive factors, MVs constitute a core molecular tool through which *S. mutans* adapts to the complex oral microenvironment, competes with other species, and initiates pathogenic processes. MVs not only regulate the balance of the local oral microecology but also serve as key mediators of intermicrobial signaling and microbe–host cross-kingdom interactions. Deciphering the pathophysiological mechanisms of *S. mutans* MVs may revolutionize caries prevention and control strategies, providing a theoretical basis for developing novel anticaries vaccines and oral targeted therapies, while also offering new research entry points for elucidating the intrinsic links between oral infections and systemic diseases.

## Key messages

- Key Message 1: *Streptococcus mutans* membrane vesicles (MVs) are actively generated through sophisticated biogenesis mechanisms involving turgor pressure, cytoplasmic membrane lipid sorting, and cell wall remodeling. Their production is precisely modulated by environmental conditions, quorum sensing signals, and genetic determinants.
- Key Message 2: *S. mutans* MVs exhibit considerable compositional heterogeneity with selective enrichment of cariogenic virulence factors, lipids, and nucleic acids, enabling them to function as critical mediators in biofilm formation, interspecies and cross-kingdom communication, and microbe-host crosstalk.
- Key Message 3: These vesicles play crucial roles in dental caries pathogenicity and systemic adverse effects while simultaneously offering promising prospects for the development of innovative anti-caries vaccines and targeted therapeutic strategies, highlighting their dual nature in disease progression and clinical translation.

## Introduction

*S* *treptococcus mutans* (*S. mutans*) is one of the predominant pathogens responsible for human dental caries. As ubiquitous constituents of the oral microbiome, *S. mutans* serves a primary function in the formation and maturation of cariogenic biofilms [1,2]. Its overall pathogenicity is primarily attributed to multiple virulence factors that drive biofilm development [3,4].

Specifically, surface adhesins mediate initial attachment of *S. mutans* to tooth surfaces and subsequent interbacterial aggregation [5,6]. Furthermore, *S. mutans* utilises glucosyltransferases (Gtfs) to metabolise dietary sucrose into exopolysaccharides (EPS), which form the fundamental three-dimensional scaffold of the biofilm matrix [7]. The active release of extracellular DNA (eDNA) further stabilises biofilm architecture through complex formation with EPS, enhancing the structural integrity and adhesive capacity of biofilms [8,9]. Beyond structural support, the pronounced acidogenicity and aciduricity of *S. mutans* enable it to act as a core pathogen within multispecies microbial communities. By lowering local environmental pH, this bacterium promotes the colonisation of other acidophilic microorganisms, such as *Candida albicans* and *Lactobacillus spp.*, ultimately fostering a dysbiotic and cariogenic oral microenvironment [10,11].

Extracellular membrane vesicles (MVs) are nanoscale lipid bilayer structures secreted by bacteria [12]. As early as the 1960s, researchers identified bioactive vesicular substances in the culture filtrates of Gram-negative bacteria, confirming their derivation from natural outer membrane budding rather than nonspecific cell lysis [13]. Historically, Gram-positive bacteria were considered incapable of producing MVs due to the thick peptidoglycan cell wall barrier. However, recent accumulating evidence has overturned this perception [14,15]. Currently, MVs have been successfully identified in a variety of Gram-positive species, including *Staphylococcus aureus*, *Bacillus anthracis*, *Enterococcus faecalis*, and *S. mutans* [16–19]. These MVs generally range from 20 to 400 nm in diameter [20]. Enriched with bioactive molecules such as lipids, proteins, and nucleic acids, bacterial MVs participate extensively in microbial metabolism, virulence regulation, interspecies communication, and microbe-host crosstalk [21–24].

In 2014, Liao et al. first isolated MVs from the culture supernatant of *S. mutans* [25]. Their work visualised the typical bilayer membrane structure of *S. mutans* MVs and clarified their key function in eDNA transportation to promote biofilm formation, pioneering research on *S. mutans*-derived MVs. Subsequent studies further verified that *S. mutans* MVs encapsulated various cariogenic virulence factors and were deeply involved in the bacterial pathogenic process [26–29]. Notably, the influence of these vesicles may extend beyond the oral cavity. A recent study by Liu et al. demonstrated that *S. mutans*-derived MVs induce dose-dependent and time-dependent impairment of endothelium-dependent vasodilation via the Akt/eNOS pathway and the p38 MAPK/endothelin-1 axis, suggesting potential systemic adverse effects [30]. Collectively, *S. mutans* MVs not only act as key regulators of local homoeostasis, mediating the remodelling of the oral microecology and driving caries progression, but also pose a potential threat to systemic health.

To date, the biogenesis, regulatory factors, cargo composition, and functional roles of *S. mutans* MVs remain incompletely understood. A comprehensive understanding of these aspects is essential for refining preventive and therapeutic strategies against dental caries and potentially systemic diseases. By systematically reviewing the literature and integrating available data, we can gain a broader perspective on the interplay between *S. mutans* MVs and oral homoeostasis, which in turn facilitates a clearer delineation of their virulence mechanisms and may open new avenues for future research. To this end, we conducted a thorough literature search in PubMed, Web of Science, and Google Scholar. The search strategy combined terms related to membrane vesicles and *S. mutans*, including ‘membrane vesicles,’ ‘extracellular vesicles,’ ‘bacterial extracellular vesicles,’ ‘microbial extracellular vesicles,’ ‘MVs,’ ‘EVs,’ ‘bEVs,’ ‘MEVs,’ ‘*Streptococcus mutans*,’ ‘*S. mutans*,’ ‘Gram-positive,’ ‘biogenesis,’ ‘cargo,’ and ‘biological functions.’ Articles published from database inception to August 2026 were considered. Original studies investigating *S. mutans* MVs were eligible, including studies about biogenesis, isolation, purification, physical characterisation, biochemical composition, cargo profiles, or biological functions of *S. mutans* MVs. Retrieved records were screened based on their titles and abstracts. Potentially relevant studies were then assessed by full-text review. Studies unrelated to *S. mutans* vesicles, review articles, conference abstracts, and reports without sufficient methodological or functional information were excluded. This review provides a comprehensive synthesis of the biogenesis, regulatory mechanisms, compositional features, and biological functions of *S. mutans* MVs. The aim is to provide novel insights and theoretical foundation for the prevention and treatment of dental caries and related systemic diseases (Figure 1).

**Figure 1.**
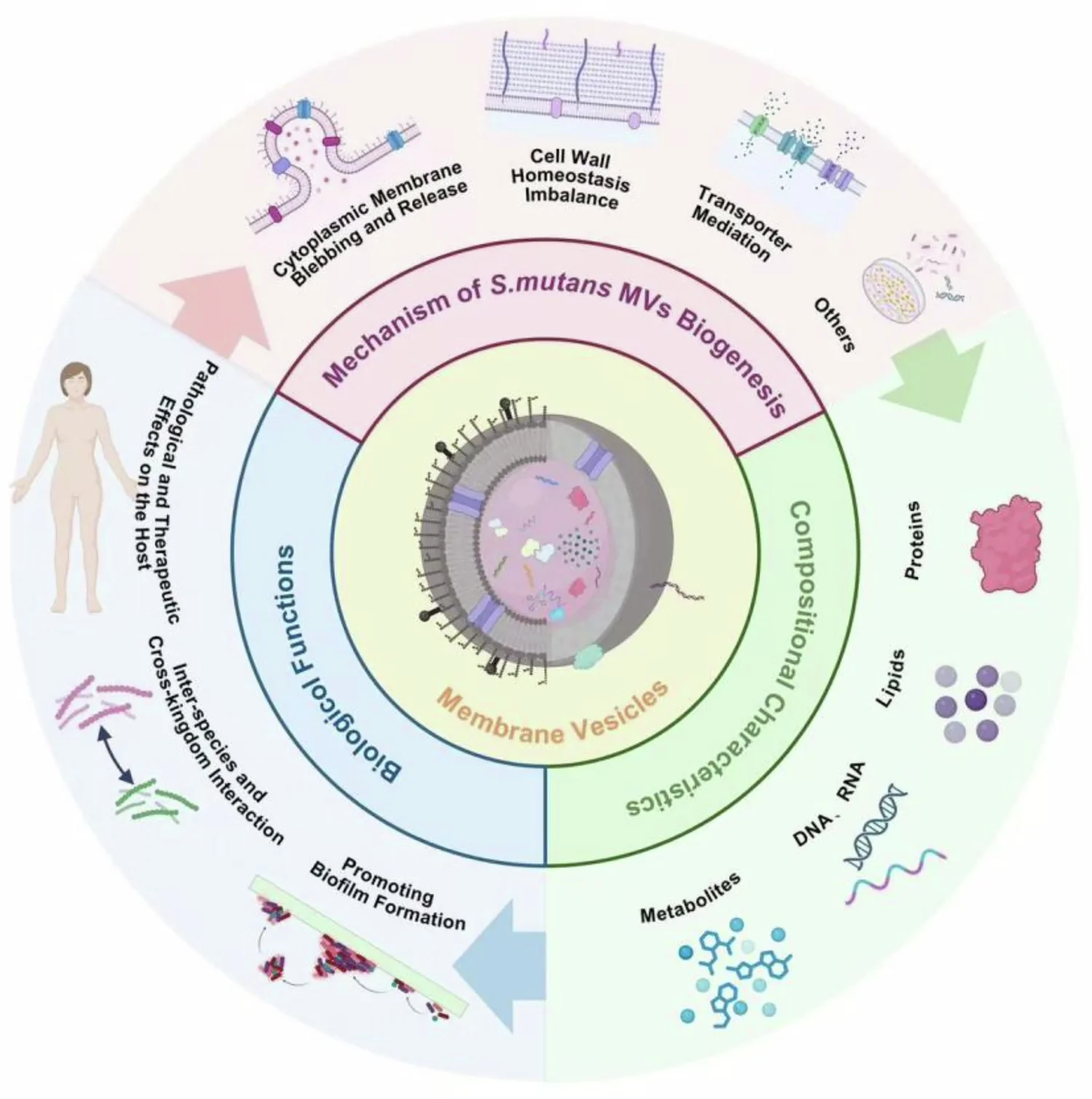
Comprehensive overview of *S. mutans* MVs: biogenesis, compositional characteristics, and multifaceted biological functions. *S. mutans* MVs originate from cytoplasmic membrane, with biogenesis driven by turgor pressure, cell wall remodelling and transporters regulation etc. They carry proteins, lipids, DNA and metabolites, promoting biofilm formation and mediating interspecies/cross-kingdom interactions, thereby contributing to cariogenicity and host crosstalk. Created in BioRender.

## Mechanism of *S. mutans* MV biogenesis

Although the biogenesis mechanism of *S. mutans* MVs remains incompletely elucidated, this sophisticated process is generally considered to arise from the combined effects of turgor pressure, cytoplasmic lipid sorting, and cell wall cross-linking status [31]. In addition, osmotic pressure mediated by specific transporters has also been proposed as a potential regulatory factor in MV biogenesis [32].

### MV Biogenesis originates from cytoplasmic membrane

Local alterations in cytoplasmic membrane fluidity together with physical pressures are fundamental determinants of MV budding. Turgor pressure acts as the major driving force that propels the extrusion of the Gram-positive bacterial cytoplasmic membrane through cell wall pores. Previous studies have demonstrated that reduced turgor pressure under hyperosmotic conditions markedly suppresses MV production in *Staphylococcus aureus*[33]. This evidence highlights the vital role of intracellular hydrostatic pressure in facilitating intracellular membrane budding toward regions with lower mechanical resistance. However, whether alterations in turgor pressure modulate the biogenesis of *S. mutans* MVs has not been thoroughly investigated and warrants future experimental confirmation.

Beyond physical factors, MV formation relies on specific lipid-sorting mechanisms. Lipidomic studies have revealed that, despite overall similarities in lipid profiles between Gram-positive bacterial cells and their secreted MVs, distinct lipid species are selectively enriched or depleted in vesicular compartments [34]. For example, *Streptococcus pyogenes* (*S. pyogenes*) MVs accumulate anionic phosphatidylglycerol while being deficient in cardiolipin [35], strongly indicating an orchestrated biogenesis process in Gram-positive bacteria. This selective lipid-sorting phenomenon has also been observed in *S. mutans* MVs [36]. Further proteomic and compositional research showed that *S. mutans* MVs contain fewer lipoproteins and integral membrane proteins [36]. Meanwhile, *srtA* knockout triggers distinct lipid remodelling in *S. mutans* MVs, including elevated abundances of glycerolipids and sphingolipids [36].

Of note, earlier investigations identified the ExPortal, an anionic lipid-rich membrane microdomain, as the primary biogenesis and secretion site of *S. pyogenes* MVs. Analogous ExPortal-like membrane compartments have also been characterised in *S. mutans*, where key MV biogenesis regulators, such as SecA and sortase A, are co-localised. This specialised microdomain modulates the secretion of major virulence factors of *S. mutans,* including cell surface protein P1 and wall-associated protein A [37,38]. In addition, *srtA* deletion alters the protein composition of *S. mutans* MVs and reduces eDNA abundance [36]. Taken together, these findings suggest that the ExPortal-like compartment functions not only as a conserved protein secretion gateway, but also as a dedicated hub that coordinates MV assembly and budding in *S. mutans* [36].

Previous studies concur that *S. mutans* MV formation is an active, regulated process involving membrane remodelling and cell-wall changes. Nevertheless, direct evidence for some proposed mechanisms is still limited in this species, and these hypotheses await further validation, including turgor‑pressure‑driven budding and ExPortal‑like biogenesis sites.

### Cell wall influences MV release

Gram-positive bacteria possess a thick cell wall (approximately 20-80 nm), which serves as the major physical barrier restricting MV release [39,40]. Accordingly, disrupting the dynamic balance of peptidoglycan synthesis and degradation, as well as increasing cell wall porosity, is essential for efficient MV secretion. The autolysin-dependent pathway is a crucial regulatory mechanism governing MV biogenesis in *S. mutans* by mediating cell wall remodelling. Through proteomic profiling, Joyce et al. identified that the AtlA autolysin, encoded by the atlA gene, is present in *S. mutans* MVs, providing strong evidence for the contribution of cell wall–modifying enzymes in vesicle biogenesis [36]. Furthermore, they constructed a Δ*srtA* strain with inactivated Sortase A. In the Δ*srtA* strain, the maturation and processing of AtlA were markedly impaired, accompanied by aberrant cell wall remodelling, increased MV size, and reduced efficiency of MV secretion [36]. These observations indicate that the SrtA-AtlA axis modulates MV formation and release via modulating cell wall structural homoeostasis.

Quorum sensing (QS) is currently recognised as an alternative and critical regulatory mechanism for MV biogenesis under specific environmental stimuli. In *S. mutans,* MV production is highly dependent on the Com system. The two-component system ComDE orchestrates a cascade signalling pathway (CSP → ComDE → CipB → ComRS → SigX → LytF) to regulate the expression of the autolysin LytF, which subsequently cleaves the peptidoglycan cell wall of *S. mutans*. This process induces autolysis and cell death in a subpopulation of cells, ultimately leading to the substantial release of MVs [41]. This mechanism underscores the adaptive ecological significance of partial programmed cell death in microbial populations.

In addition, the endolysin-related pathway has emerged as a novel modulator of Gram-positive MV biogenesis. Under stress conditions such as DNA damage (SOS response), prophage-encoded endolysins are activated to trigger MV production through a mechanism distinct from canonical endolysin-related explosive lysis [42]. Existing evidence demonstrates that endolysins in typical Gram-positive species (e.g. *Bacillus subtilis*) etch peptidoglycan in a localised and controllable pattern [42]. Such partial cell wall etching generates discrete pores that allow MV extrusion without causing immediate bacterial lysis. Whether this conserved endolysin-dependent MV biogenesis mechanism is functional in *S. mutans* remains unclarified. Nevertheless, this pathway provides a promising new perspective for understanding stress-triggered MV release in *Streptococci*.

### The transporter mediates MV release

Environmental stress, particularly acidic conditions, indirectly modulates MV release by altering the functional activity of membrane transporters [32,43]. Previous studies have demonstrated that the ABC transporter OpuB contributes substantially to the maintenance of membrane homoeostasis in *S. mutans* [43]. Under acid stress, the *Smu_opuB* mutant strain exhibits impaired membrane homoeostasis and aberrant MV secretion [32], suggesting that OpuB preserves intracellular turgor pressure by fine-tuning osmotic balance across the bacterial membrane. Upon OpuB dysfunction, compensatory stress-responsive pathways are activated, which compromise membrane integrity and consequently trigger non-specific, stress-induced MV release.

### Factors affecting *S. mutans* MV biogenesis

The biogenesis of *S. mutans* MVs is not a stochastic process. Instead, it is an active, finely tuned event governed by bacterial growth conditions, signal transduction pathways, and genetic backgrounds.

#### Growth conditions

Physicochemical growth parameters serve as fundamental determinants that modulate the biogenesis and functional properties of *S. mutans* MVs. Initial environmental pH shapes not only physical features of *S. mutans* MVs, including particle size and production yield, but also profoundly remodels their cargo profiles. Under neutral conditions (pH 7.5), *S. mutans* tends to secrete fewer MVs with larger sizes [43]. In contrast, acidic environments (pH 5.5) promote the production of smaller *S. mutans* MVs at higher yields [43]. Despite a marked reduction in total protein abundance within these small-sized *S. mutans* MVs, their cargo undergoes a selective remodelling process: 20 proteins are markedly upregulated, while 120 others are downregulated. Such specific alterations in vesicular cargos strengthen the capacity of *S. mutans* MVs to facilitate biofilm formation and acid production [43]. Interestingly, *S. mutans* exhibits a similar trend under oxygen stress. Although a normoxic environment (21% O_2_) is unfavourable for its growth and reproduction, with reduced colonisation and adhesion, decreased biofilm mass and consequently lower cariogenic potential, the secretion of MVs is 100 times higher than under anaerobic conditions (5% CO_2_). Moreover, these MVs are enriched in the adhesion-related proteins GtfC and SpaP, which enhance glucan synthesis, bacterial self-aggregation, and surface adhesion, thereby promoting initial adhesion and early biofilm formation of *S. mutans*. As such, during enamel demineralisation, *S. mutans* not only possesses resistance mechanisms to cope with acidic pH changes but also releases abundant MVs to alleviate acid and oxygen stress, enhancing its survival advantage within plaque biofilms [44].

Beyond extracellular pH fluctuations, medium composition profoundly shapes bacterial metabolism and consequently governs MV characteristics and yield by modulating QS pathways. For instance, culture medium regulates quorum sensing in *S. mutans* in a pathway-selective manner, thereby influencing the production of MVs. In complex media such as brain heart infusion (BHI), the CSP-ComDE signalling pathway is readily activated. In contrast, in chemically defined medium (CDM), the ComRS-XIP pathway dominates the generation of MVs, while the activity of the CSP pathway is markedly restricted [41]. Functional complementation assays further revealed that exogenous MVs derived from BHI-cultured *S. mutans* UA159 could fully rescue the biofilm formation deficiency of the *gtfBC*-deficient strain, whereas CDM-derived MVs exhibited only marginal rescuing capacity [41]. These findings confirmed that medium composition may serve as a decisive factor, influencing MV biogenesis and thereby affecting the biological functions of *S. mutans* MVs.

Bacterial growth phase and culture model also exert substantial impacts on MV biogenesis. Cui et al. reported that MVs harvested from mid-logarithmic-phase *S. mutans* (BHI culture) significantly inhibited biofilm formation of *Streptococcus gordonii* DL-1 [45]. Conversely, Senpuku et al. observed opposing results using *S. mutans* MVs cultured in TSB medium against *Streptococcus gordonii* ATCC10558 [46]. Such inconsistent observations are most likely attributable to variations in bacterial strains, medium formulas, and incubation durations, emphasising the necessity of standardised culture conditions for future investigations regarding growth-dependent MV regulation. Regarding spatial growth modes, planktonic *S. mutans* produce the largest MVs, while bacteria adhered to the collagen surface secrete vesicles of intermediate size, and biofilm-embedded *S. mutans* generate the smallest MVs [28].

Emerging evidence has highlighted the regulatory role of host pathological microenvironments in modulating bacterial MV production. By mimicking the glycated collagen microenvironment characteristics of diabetic patients, in vitro glycosylated collagen substrates notably reduce the yield and recovery efficiency of *S. mutans* MVs [28]. This phenomenon indicates that host pathological alterations, such as matrix glycosylation, directly reshape bacterial vesicle biogenesis, pathogenic potency, and interbacterial communication. These findings provide novel insights into the complex host-microbe interactive mechanisms under diseased oral microenvironment.

#### Peptide signals

The Com system critically modulates autolysin-dependent MV biogenesis in *S. mutans*. Two peptide signalling molecules, CSP and XIP, separately activate the ComDE and ComRS pathways. These pathways synergistically upregulate the transcription of the alternative Sigma factor gene *sigX* and its downstream autolysin gene *lytF*, thereby facilitating *S. mutans* MV release [41]. The exogenous supplementation with CSP or XIP not only elevates *S. mutans* MV production yield, but also reshapes the selective cargo packaging and biological functions of secreted MVs [41,47].

#### Bacterial strains and gene regulation

Genetic variation among *S. mutans* strains substantially alters MV biogenesis efficiency. Clinical isolates with distinct virulent backgrounds exhibit highly different vesicle production profiles. For instance, the cariogenic clinical strain *S. mutans* 27-3 releases approximately eight-fold more MVs than the standard laboratory strain UA159, suggesting that MV secretion capacity is strongly correlated with strain-specific virulence potential. In particular, comparative genomic analysis revealed that, unlike UA159, strain 27-3 naturally lacks the *bacA* gene, a feature that might explain why MV production is greatly increased [48]. In addition, multiple genetic loci have been verified as crucial genetic determinants governing *S. mutans* MV formation and secretion.

The cyclic di-AMP (c-di-AMP) signalling pathway serves as a core regulatory module regulating *S. mutans* MV secretion. Knockout of c-di-AMP metabolism-related genes, including *dac* and *pdeA*, significantly elevates *S. mutans* MV production [48]. Consistently, deletion of *bacA*, a surfactin synthetase homologue located within the TnSmu2 genomic island, impairs c-di-AMP synthesis and consequently enhances vesicle biogenesis [48]. Such alterations are likely attributed to c-di-AMP fluctuation, which widely affects bacterial membrane homoeostasis, biofilm architecture, and acid tolerance. In addition, deletion of the TnSmu2-encoded *sfp* gene could also increase *S. mutans* MV yield, alter vesicle stability, and compromise bacterial resistance to acid and oxidative stress [48]. Intriguingly, deletion of another homologous bacitracin synthetase gene *bacA2* results in a nearly 50% reduction in *S. mutans* MV production [48]. Collectively, these evidences demonstrate that the TnSmu2 genomic island exerts complex and bidirectional regulatory effects on *S. mutans* MV biogenesis through distinct genetic components.

Autolysin-mediated cell wall remodelling also constitutes an essential pathway for MV biogenesis in *S. mutans* [41]. Genetic ablation of autolysin-encoding genes distinctly reduces vesicle production, and this defective phenotype can be functionally rescued by exogenous *lytF* complementation [41]. Mechanistically, activation of the Com system upregulates *lytF* expression, which promotes localised cell wall hydrolysis and partial subpopulation cell death, thereby facilitating *S. mutans* MV budding and release. Beyond autolysin-related machinery, the Gtf-encoding gene *smu_833* acts as a negative regulator of vesicle biogenesis. *smu_833* deletion considerably increases the secretions of both *S. mutans* MVs and eDNA, possibly by compromising cell wall integrity [27].

Beyond modulating MV biogenesis, specific genes predominantly shape the compositional characteristics and physicochemical properties of *S. mutans* MVs. For example, the Gtf-encoding genes for extracellular glucan synthesis, also significantly regulate the protein cargo and particle size of *S. mutans* MVs [49]. Deletion of these genes impairs the capacity of *S. mutans* MVs to promote biofilm formation [49]. The ABC transporter gene *opuB*, which participates in osmotic adaptation and environmental stress responses, also modulates vesicle phenotypes. Under neutral conditions, *opuB* deficiency leads to the production of more but smaller *S. mutans* MVs, accompanied by distinct lipid remodelling: the abundance of acyl carnitine (AcCa) is elevated, whereas lysophosphatidylethanolamine (LPE) content is reduced in these vesicles [32]. Moreover, deletion of the transpeptidase-encoding gene *srtA* does not suppress *S. mutans* MV yield, but significantly alters vesicle size and protein profiles. Specifically, the abundance of major adhesive proteins, including adhesin P1, is markedly decreased in *S. mutans* MVs derived from the *srtA*-deficient strain [36]. To date, all documented genes associated with *S. mutans* MV modulation are summarised in Table 1.

**Table 1.** Role of specific *S. mutans* genes in MV biogenesis.

Gene	Protein	Influence of Gene Deletion on MV size	Influence of Gene Deletion on MV Production	Influence of Gene Deletion on MV Content Composition	Reference
*dac*	Dac	/	increased	/	[48]
*pdeA*	PdeA	/	increased	/	[48]
*bacA*	Bac	/	increased	/	[48]
*bacA2*	BacA2	/	decreased	/	[48]
*sfp*	Sfp	/	increased	/	[36,48]
*lytF*	LytF	/	decreased	/	[41]
*smu_833*	Smu_833	/	increased	/	[27]
*gtfB*	GtfB	/	/	protein content: decreased; biofilm formation: decreased	[49]
*gtfC*	GtfC	decreased	/	protein content: decreased; biofilm formation: decreased	[49]
*opuB*	OpuB	Decreased (neutral condition)	Increased (neutral condition)	lipids (such as AcCa): increased; lipids (such as LPE): decreased	[32]

However, current investigations regarding genetic modulation of *S. mutans* MV structure and function remain largely descriptive. The precise molecular mechanisms, as well as the upstream and downstream pathways involved in these genetic networks, remain poorly defined. Further mechanistic studies are required to elaborate the comprehensive signalling pathways governing *S. mutans* MV compositional and functional remodelling.

Genetic studies consistently show that specific genes involved in cell wall remodelling, membrane homoeostasis, and stress responses can regulate formation of *S. mutans* MVs. Deletion of different genes can either increase or decrease MV production, suggesting the involvement of multiple regulatory pathways rather than a single common mechanism. However, the relationships among these pathways remain poorly defined, and systematic investigations of the underlying gene regulatory networks are still warranted.

In summary, the biogenesis of *S. mutans* MVs involves core mechanisms such as turgor pressure, lipid microdomain sorting, and cell wall remodelling, and is modulated by various factors: environmental conditions, peptide signals, strain variations, and genetic regulators (Figure 2). However, discrepancies among published reports may stem not only from biological regulation but also from differences in experimental design. Culture conditions, including medium composition, pH, and growth phase, may alter both *S. mutans* MV release and cargo packaging. Therefore, comparisons of MV production or functionality across studies should take these biological and experimental variables into account. As summarised in Table 2, studies have also varied considerably in their methods for MV isolation, purification, and characterisation. Most employed centrifugation, filtration, and ultracentrifugation, whereas density‑gradient purification was used in only a few reports. Physical and biochemical analyses showed substantial heterogeneity across studies. Such methodological variability may influence MV yield, purity, size distribution, and cargo profiles, thereby affecting the reported biological effects. In particular, normalisation based only on protein concentration may not accurately reflect MV abundance when the protein content per vesicle differs between strains or culture conditions. Collectively, these methodological differences should be considered when interpreting reported MV cargo compositions and functional outcomes.

**Table 2.** Comparison of methodological characteristics and their limitations of original studies on *S. mutans*.

Reference	Strains	Growth Conditions	Isolation methods for *S. mutans* MVs	Physical characterisation methods for *S. mutans* MVs	Biochemical analysis of *S. mutans* MVs	Limitations
Liao et al. [25]	*S. mutans* UA159, *S. mutans* NG8 and their derivatives	FMC containing glucose; 37 °C, 5% CO₂, static; harvested at early exponential phase (OD_600_ = 0.3) or after overnight culture	Centrifugation at 6,000 × *g* for 10 min at 4 °C; sequential 0.45-μm and 0.22-μm filtration; ultracentrifugation at 100,000 × *g* for 1 h at 4 °C; PBS wash.	TEM and cryo-EM	BCA protein assay; SDS–PAGE; western blotting; MV-associated eDNA quantification	MV preparations lacking density-gradient purification, potentially containing co-isolated soluble proteins and free nucleic acids.
Senpuku et al. [46]	*S. mutans* UA159, *S. mutans* UA159 *gtfBC* mutant	BHI; 37 °C, 5% CO_2_; 24 h	Centrifugation at 6,000 × *g* for 20 min; > 10- or > 50-kDa centrifugal concentration; sequential 0.45- and 0.22-μm filtration; ultracentrifugation at 150,000 × *g* for 2 h.	/	Native PAGE and SDS–PAGE; western blotting; complex-DNA analysis	Difficulties in separating intact MVs from co-isolated GTF–DNA complexes due to limited purification and the lack of direct particle characterisation.
Rainey et al. [27]	*S. mutans* UA159, *S. mutans* Δ*smu_833* and the complemented strains	FMC containing glucose; 37 °C, 5% CO_2_, static; 16 h	Centrifugation at 6,000 × *g* for 10 min at 4 °C; 0.22-μm filtration; ultracentrifugation at 140,153 × *g* for 2 h at 4 °C; PBS wash.	/	BCA protein assay for MV yield; qPCR	MV morphology and cargo composition not comprehensively characterised.
Morales-Aparicio et al. [36]	*S. mutans* NG8, *S. mutans* NG8 Δ*srtA*, *S. mutans* PC339, *S. mutans* PC339 Δ*sfp*	Overnight preculture in 40 ml Todd–Hewitt broth containing 3% yeast extract, then 10 mL was transferred to 1 L of TDM and grown at 37◦C for 16 h.	Centrifugation at 18,000 × *g* for 30 min at 4 °C; 0.2-μm filtration and 100-kDa ultrafiltration; ultracentrifugation at 100,000 × *g* for 1 h at 4 °C; OptiPrep gradient centrifugation at 140,000 × *g* for 16 h at 4 °C; final centrifugation at 100,000 × *g* for 2 h at 4 °C.	TEM, NTA, DLS and *ζ*-potential	SDS–PAGE and western blotting; Impact II QTOF LC-MS/MS for analysis of lipids; Q Exactive HF Orbitrap LC-MS/MS for analysis of proteins	Limited genetic background, untested functional effects of protein/lipid changes in lab mutants.
Cao et al. [43]	*S. mutans* UA159	BHI at initial pH 7.5 (8 h) or 5.5 (12 h); 37 °C, anaerobic (80% N_2_,10% CO_2_ and 10% H_2_); harvested at mid-exponential phase	Sequential centrifugation at 6,000 × *g* for 15 min and 10,000 × *g* for 15 min at 4 °C; 0.22-μm filtration and 100-kDa tangential-flow filtration; ultracentrifugation at 100,000 × *g* for 70 min at 4 °C; OptiPrep gradient centrifugation at 200,000 × *g* for 3 h at 4 °C; final centrifugation at 100,000 × *g* for 70 min at 4 °C.	TEM and NTA	BCA protein assay; label-free quantitative proteomics; bioinformatic analysis; biochemical assays; untargeted metabolomics by mass spectrometry	One strain, two pH conditions, descriptive omics, and unresolved cargo causality.
Wu et al. [50]	*S. mutans* UA159	BHI; 37 °C, 16 h	Sequential centrifugation at 6,000 × *g* for 15 min and 10,000 × *g* for 15 min at 4 °C; 0.22-μm filtration and 100-kDa ultrafiltration; ultracentrifugation at 100,000 × *g* for 70 min at 4 °C.	TEM and NTA	BCA protein assay	Crude ultracentrifugation preparations and protein-based dosing may include non-vesicular material, and the specific MV cargo responsible for the effects on *C. albicans* remains unidentified.
Nakamura et al. [49]	*S. mutans* UA159, UA159 Δ*gtfB*, UA159 Δ*gtfC* and UA159 Δ*gtfBC*	BHI; 37 °C, 5% CO_2_; 24 h	Centrifugation at 6,000 × *g* for 20 min; > 50-kDa centrifugal concentration; sequential 0.45- and 0.22-μm filtration; ultracentrifugation at 150,000 × *g* for 2 h at 4 °C, performed twice.	SEM	SDS–PAGE and western blotting	MV size and morphology were assessed mainly by SEM, without comprehensive particle-size analysis.
Wen et al. [48]	*S. mutans* wild-type UA159 and its derivatives, including clinical strain 27-3	FMC containing 20 mM glucose; 37 °C, 5% CO_2_, static; overnight	Centrifugation at 6,000 × *g* for 10 min at 4 °C; sequential 0.45- and 0.22-μm filtration; ultracentrifugation at 100,000 × *g* for 90 min at 4 °C; PBS wash.	TEM; flow cytometry of FM1–43-labelled MV particles	BCA protein assay; MV-associated eDNA quantification	Estimation of MV yield by protein/fluorescent‑particle measurements, and largely undefined cargo selection and individual cargo functions.
Iwabuchi et al. [26]	*S. mutans* UA159, UA159 Δ*gtfB*, UA159 Δ*SMU833*, UA159 Δ*sacB*	BHI at initial pH 6.0, 7.4 or 8.0; 37 °C, 5% CO_2_; overnight	Centrifugation at 6,000 × *g* for 20 min; > 50-kDa centrifugal concentration; sequential 0.45- and 0.22-μm filtration; ultracentrifugation at 150,000 × *g* for 2 h at 4 °C, performed twice.	SEM	SDS–PAGE and western blotting; zymography for Gtf activity; peptide mass fingerprinting (PMF) Analyses of MVs; qPCR	Potential medium-specific effects from pH adjustment, limited quantitative resolution of SEM sizing, and unestablished pH-responsive cargo.
Cui et al. [45]	*S. mutans* UA159, UA159 Δ*gtfB*	BHI; 37 °C, anaerobic (80% N_2_, 10% H_2_, and 10% CO_2_); 16 h.	Sequential centrifugation at 6,000 × *g* for 15 min and 10,000 × *g* for 15 min at 4 °C; 0.22-μm filtration; 100-kDa ultrafiltration at 4,000 × *g* for 30 min at 4 °C; ultracentrifugation at 100,000 × *g* for 70 min at 4 °C, performed twice.	/	BCA protein assay	Total protein-normalised MVs examined exclusively in vitro, without direct cargo profiling or mixed-species/in vivo validation.
Wu et al. [51]	*S. mutans* UA159	*S. mutans* in BHI; 37 °C; 16 h	Sequential centrifugation at 6,000 × *g* for 15 min and 10,000 × *g* for 15 min at 4 °C; 0.22-μm filtration and 100-kDa ultrafiltration; ultracentrifugation at 100,000 × *g* for 70 min at 4 °C.	/	BCA protein assay	In vitro bovine-dentin model, with *S. mutans* MV cargos neither directly isolated nor validated.
Song et al. [52]	*S. mutans* ATCC 25157	BHI, 37 °C for 16 h	Centrifugation at 8,000 × *g* for 20 min at 4 °C; 0.22-μm filtration; 100-kDa ultrafiltration at 4,000 × *g* for 30 min at 4 °C; ultracentrifugation at 100,000 × *g* for 90 min at 4 °C, followed by two additional wash centrifugations (parameters not specified).	/	BCA protein assay	Protein-quantified MVs with uncharacterised size, purity, and cargo.
Mizuta and Suzuki [53]	*S. mutans* UA159, UA159 Δ*gtfBC*	BHI, 37 °C, 5% CO_2_; overnight	Ultrafiltration at 3,000–4,000 × *g* for 20 min at 4 °C; sequential 0.45- and 0.22-μm filtration; ultracentrifugation at 150,000 × *g* for 2 h at 4 °C.	/	Bio-Rad protein assay	MV quantification based solely on total protein, lacking morphological, size, and purity assessment, with the roles of GtfB/GtfC and eDNA inferred from mutant and DNase experiments.
Leiva-Sabadini et al. [28]	*S. mutans* UA159	BHI; 37 °C, aerobic; 24 h	Centrifugation at 4,000 rpm for 20 min at 4 °C; 0.22-μm filtration; ultrafiltration at 4,000 rpm for 15 min at 4 °C; ultracentrifugation at 125,000 × *g* for 2 h at 4 °C.	AFM, NTA and electron microscopy	Protein quantification; quantitative LC–MS/MS proteomics	Static mono-species biofilm model lacking oral complexity, descriptive proteomics without causal cargo assignment, and no individual deletion, depletion, or reconstitution experiments to test biofilm or virulence effects.
Nagasawa et al. [41]	*S. mutans* UA 159 and mutants in *comCDE*, *cipB*, *comRS*, *sigX* and *lytF*	BHI or CDM; overnight cultures were diluted to an OD_600_ of 0.01 and cultured in an aerobic atmosphere (5% CO_2_) at 37 °C for 24 h. sCSP and sXIP were added at the start of culture at final concentrations of 1 μM and 10 nM, respectively.	Centrifugation (parameters were not specified), followed by 0.45-μm PVDF filtration; ultracentrifugation at 150,000 × *g* for 1 h at 4 °C; resuspension in PBS; further purification by 45%–10% iodixanol density-gradient ultracentrifugation (force and duration were not specified).	SEM and TEM	FM®1-43FX fluorescence-based CMV quantification; SDS–PAGE; band identification by MALDI–TOF/MS and MASCOT database search.	Artificial CSP/XIP stimulation with limited fidelity to oral biofilm signalling, and unresolved vesicle origin (active release versus cell lysis).
Wang et al. [32]	*S. mutans* UA159, *Smu_opuB*	BHI at initial pH 7.5 or 5.5; 37 °C, anaerobic; harvested at mid-log phase (OD_600_ = 0.2–0.3); exact duration not reported	Sequential centrifugation at 6,000 × *g* for 15 min and 10,000 × *g* for 15 min at 4 °C; 0.22-μm filtration and 100-kDa tangential-flow filtration; ultracentrifugation at 100,000 × *g* for 70 min at 4 °C; OptiPrep gradient centrifugation at 200,000 × *g* for 3 h at 4 °C.	TEM analysis and nanoflow cytometry.	BCA protein assay; quantitative proteomics; lipidomics	Broth-culture duration not reported; pH-associated proteomic and lipidomic changes remain unvalidated mechanistically and in physiological models.
Iwabuchi et al. [54]	*S. mutans* UA159, UA159 Δ*gtfBC*	Bacteria were cultivated with various concentrations of NaCl in tryptic soy broth with 0.25% sucrose or 0.25% glucose for 16 h. NaCl was applied at nine concentrations ranging from 0.0039 to 1.0 M using a twofold serial-dilution series, with a no-NaCl control included.	Centrifugation at 6,000 × *g* for 20 min; > 50-kDa centrifugal concentration; sequential 0.45- and 0.22-μm filtration; ultracentrifugation at 150,000 × *g* for 2 h at 4 °C, performed twice.	/	Bio-Rad protein assay	MV preparations uncharacterised here, validated by prior TEM/SEM, and lacking density-gradient purification, with possible non-MV co-isolation.
Oh et al. [55]	*S. mutans* (KCTC 3065, ATCC 25175)	BHI containing vitamin K and hemin; 37 °C, aerobic; harvested at OD_600_ = 1.5; exact duration not reported	Centrifugation at 6,000 × *g* for 20 min; 0.22-μm filtration and 100-kDa tangential-flow filtration; ultracentrifugation at 100,000 × *g* for 2 h at 4 °C.	TEM and NTA	Isolation of EV-associated RNA; RNA quality analysis by Bioanalyzer; small-RNA sequencing for tRNA-cargo profiling	RNA cargo characterised, protein/lipid unanalysed, and RNA species contribution requiring further validation.
Hashizume-Takizawa et al. [56]	*S. mutans* UA159 and UA159 Δ*gtfBC*	BHI; 37 °C, 5% CO_2_; 24 h	Centrifugation at 6,000 × *g* for 20 min; > 50-kDa ultrafiltration; sequential 0.45- and 0.22-μm filtration; ultracentrifugation at 150,000 × *g* for 2 h, performed twice.	SEM	SDS–PAGE and western blotting	Particle characterisation was limited to SEM, with immunogenicity not evaluated in caries or long-term safety models.
Zhang et al. [57]	*S. mutans* UA159	BHI; 37 °C, 5% CO_2_; 16 h	Sequential centrifugation at 6,000 × *g* for 15 min and 10,000 × *g* for 15 min at 4 °C; 0.22-μm filtration; ultracentrifugation at 100,000 × *g* for 70 min at 4 °C; PBS wash.	TEM and NTA	BCA protein assay	Mechanisms by which MVs regulate *C. albicans* ubiquitination require further elucidation.
Boone et al. [58]	*S. mutans* UA159	Overnight BHI preculture, followed by BHI containing 1% sucrose at pH 7.2 or 5.2; 37 °C, aerobic with agitation; overnight	Sequential centrifugation at 3,500 × *g* for 15 min and 10,000 × *g* for 15 min at 4 °C; 0.22-μm filtration; ultracentrifugation at 100,000 × *g* for 70 min at 4 °C. For selected TEM samples only, OptiPrep gradient centrifugation at 140,000 × *g* for 19 h at 4 °C.	TEM and NTA	DC protein assay; SDS–PAGE with silver staining; small-RNA isolation and Qubit/Bioanalyzer quality assessment; small-RNA sequencing	Crude ultracentrifugation-based RNA analysis, with RNA functions inferred but not experimentally validated.
Kim et al. [59]	*S. mutans* (KCTC 3065, ATCC 25175)	BHI containing vitamin K and hemin; 37 °C, aerobic; harvested at OD_600_ = 1.5; exact duration not reported	Centrifugation at 6,000 × *g* for 20 min at 4 °C; 0.22-μm filtration; ultracentrifugation at 100,000 × *g* for 2 h at 4 °C.	TEM and NTA	Protein quantification	MV cargo unidentified, with no biochemical profiling or in vivo validation.
Liu et al. [30]	*S. mutans* (ATCC 35668)	TSA plates; 37 °C, aerobic; 24 h, followed by culture in 5 mL BHI broth to approximately 10^9^ CFU/mL; broth-culture duration was not reported.	Sequential centrifugation at 3,000 × *g* for 10 min and 10,000 × *g* for 30 min at 4 °C; 0.22-μm filtration; ultracentrifugation at 120,000 × *g* for 70 min at 4 °C, performed twice.	TEM and NTA	Western blotting; silver staining of MV proteins	Unidentified *S. mutans* MV cargo underlying endothelial dysfunction, unclear mechanisms of barrier translocation and uptake, and unquantifiable EV dosing limiting in vitro and in vivo modelling.
Cao et al. [4]	*S. mutans* UA159	BHI; 37 °C, anaerobic (5% CO_2_, 10% H_2_, and 85% N_2_); overnight.	Sequential centrifugation at 6,000 × *g* for 15 min and 10,000 × *g* for 15 min at 4 °C; 0.22-μm filtration; 100-kDa tangential-flow filtration; ultracentrifugation at 100,000 × *g* for 70 min at 4 °C.	TEM and NTA	BCA protein assay	No density-gradient purification or cargo analysis, a static mono-species biofilm model, and unidentified MV components or surface properties underlying enhanced early biofilm formation.
Dufour et al. [47]	*S. mutans* UA159, UA159 Δ *pep299*, and *pep299*-overexpressing strain	Todd-Hewitt yeast extract; 37 °C, 5% CO_2_; Overnight cultures (20 h) were diluted 1:100 into fresh THYE with or without 50 ng/mL CSP and harvested at logarithmic or stationary phase.	Centrifugation at 4,000 × *g* for 15 min at 4 °C; 0.22-μm filtration; 100-kDa ultrafiltration; ultracentrifugation at 140,000 × *g* for 1 h at 4 °C.	NTA	BCA protein assay; LC-MS/MS proteomics; R18 dequenching assay for MVs-cell fusion.	No density-gradient purification, examination limited to Pep299, and the roles of other CSP-responsive MV cargos in antibiotic persistence remain unclear.
Cui et al. [44]	*S. mutans* UA159 and UA159 Δ*gtfBC*	BHI; 37 °C. Cultures were grown overnight (approximately 12 h) under normoxic conditions (21% O₂, aeration) or hypoxic conditions (5% CO_2_) before MV collection.	Sequential centrifugation at 6,000 × *g* for 15 min and 10,000 × *g* for 15 min at 4 °C; 0.22-μm filtration; 100-kDa ultrafiltration at 3,500 × *g* for 30 min at 4 °C; ultracentrifugation at 100,000 × *g* for 70 min at 4 °C, followed by a PBS wash and a second ultracentrifugation.	TEM, NTA and nanoflow cytometry	BCA protein assay; qualitative and quantitative proteomic analyses; untargeted metabolomics analysis.	Ultracentrifugation without density-gradient purification, with possible co-isolation of non-MV material; GtfC/SpaP were enriched in normoxic MVs, but their roles in adhesion and biofilm formation remain unconfirmed.

**Figure 2.**
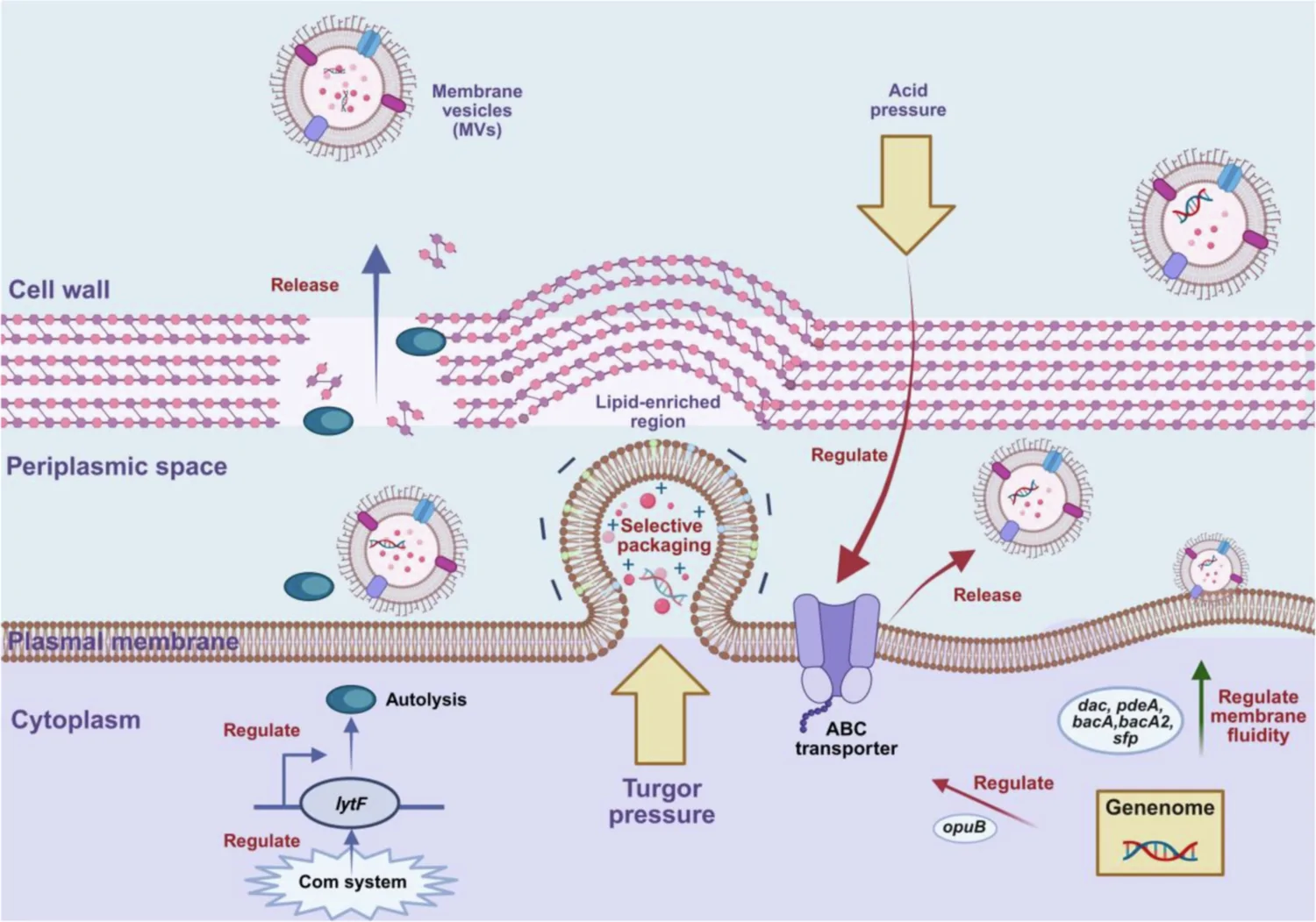
Mechanism and Factors Modulating *S. mutans* MV biogenesis. MV formation in bacteria is an active, rather than passive, process. It is precisely controlled by sophisticated gene regulatory networks and influenced by environmental signals, leading to the disruption of cell wall dynamic homoeostasis and subsequently promoting MV biogenesis and release. Created in BioRender.

## Compositional characteristics

MVs produced by *S. mutans* display a complex, highly dynamic and heterogeneous composition, containing proteins, lipids, and nucleic acids. These cargos can mediate intraspecies, interspecies, and even cross-species communication, in which they exert diverse biological functions. They play critical roles in eliciting immune responses, mediating horizontal gene transfer, facilitating nutrient acquisition, and delivering virulence factors [60]. Consequently, the functions of MVs are largely determined by their cargo composition.

### Proteins

Proteins constitute the primary functional cargo that mediates the biological activities of *S. mutans* MVs. MVs derived from *S. mutans* are highly enriched in key cariogenic virulence factors, including Gtfs, SpaP (also known as Pac, Antigen I/II, P1 or MSL-1), and glucan-binding proteins, rendering MVs pivotal vehicles for the extracellular translocation and distal delivery of pathogenic factors. Quantitative proteomics has identified a total of 509 proteins packaged within *S. mutans* MVs under different pH conditions, among which 344 core proteins are constitutively conserved at both pH 5.5 and 7.5 [43]. Nevertheless, specific genetic manipulations substantially reshape vesicular protein profiles. For instance, deletion of the transpeptidase gene *srtA* considerably alters the protein repertoire of *S. mutans* MVs [36]. These findings strongly demonstrate that protein sorting into *S. mutans* MVs is a highly regulated, selective biological process rather than a passive or random incorporation event and the protein composition of *S. mutans* MVs differs under different environmental and genetic conditions (Table 3).

**Table 3.** Upregulated and downregulated proteins in *S. mutans* MVs under different conditions.

Condition	Upregulated proteins	Downregulated proteins	Reference
Proteins in *S. mutans* MVs cultured at pH 5.5 compared to pH 7.5	GbpD, GbpA, GtfB, GtfD, TpiA, Pgk, LeuS, Gap, SMU_689, RelA, PfkA, KxYKxGKxW signal peptide-containing protein, GapC, putative hydrolase SMU_367, IlvC, GapA_2, LysS	GtfC, LDH, DexA, Pbp1a, membrane carboxypeptidase, Pbp2a, PTS-associated proteins, GroEL, and DnaK	[43]
Proteins in *S. mutans* MVs cultured at biofilm conditions (both glycated and non-glycated collagen) compared to planktonic *S. mutans* MVs	RpmA, Pnp, GroES, TrxB, DapA, GshR, GalE, AtpA, TyrS, NifS, ThyA, Tpx, Ung, FabH, SMU_1040c, GreA, SSB, LivF, Clp, SMU_1788c, GroEL, Ffh, LuxS, NusG, SMU_1193, TreA, SMU_229, SMU_984, PGM (SMU_1106c), SMU_399, Pgi, SMU_1238c, RplK	/	[28]
Proteins in *S. mutans* MVs from control collagen-bound biofilms compared to *S. mutans* MVs from planktonic bacteria	Enolase, Dpr, PtxB, YidC1, YidC2, MltG, SrtA, DltA, DltC, ClpX, ScrA	/	[28]
Proteins in *S. mutans* MVs cultured at pH 8.0 compared to pH 7.4	GtfB, GtfC	/	[26]
Proteins in *S. mutans* WT MVs compared with corresponding cytoplasmic membrane	SMU_1904c, PotD, SMU_367, FruA, GbpD, Ftf, GbpC, GbpB, AtlA, SMU82_1213c, GtfC, SpaP, GtfB, SMU_63c, DexA, GtfD, SMU_1733c, SMU_609	/	[36]
Proteins in *S. mutans* Δ*srtA* MVs compared with corresponding cytoplasmic membrane	SMU_1904c, GbpC, Ftf, AtlA, GbpB, GtfC, DexA, SMU82_1213c, GtfB	/	[36]
Proteins in *S. mutans* Δ*sfp* MVs compared with corresponding cytoplasmic membrane	SMU_963c, SMU_1904c, SMU_172, SMU_367, BacA2, BacA, WapE, FruA, GbpD, GbpB, AtlA, GbpC, Ftf, GtfD, SMU_63c, GtfC, SMU82_1213c, GtfB, BrpA	/	[36]
Proteins in *S. mutans Smu_opuB* MVs compared with *S. mutans* UA159 at pH 7.5	SMU_1344c, ErmB, A6J86_004325, NagB, SMU_1205c, SMU_459, SpaP, RplO, DexA, RpsK	SMU_438c, EF-Ts, SMU_936, SMU_1118c, PrmA, SloR, SMU82_02671, SMU_923, SMU_728, SMU_922	[32]
Proteins in *S. mutans Smu_opuB* MVs compared with *S. mutans* UA159 at pH 5.5	SMU82_03911, ErmB, SMU_1463c, AlsS, Rpe, NagB, HisD, AroK, NrdG, SMU_1584c	SMU_1171c, A6J86_008015, SMU_1078c, SMU_922, MetAA, SMU_237c, PstA, SMU82_04358, MbrB, FtsL	[32]
Proteins in *S. mutans* MVs from CSP-induced cultures compared with non-induced cultures at the stationary phase	Pep299, Dpr, TrxB, Tpx, MsrA, SMU.328, CitM, SatE, YlxM, SMU.1289, MpfA, OpuBB, SMU.1215, MutS2, SMU.2087, YidC1, LiaF, NlmB, MutE2, BrsR, and other proteins associated with stress, antimicrobial and persistence (233 proteins enriched)	37 proteins, mainly cytoplasmic and membrane-associated proteins	[47]
Proteins in *S. mutans* MVs under normoxic conditions compared with hypoxic conditions	GtfC, SpaP, GtfB, Ftf, MurD, AtlA, GlgC, SpxA, GroEL, and proteins involved in transport, proteolysis, glycolysis, peptidoglycan biosynthesis, extracellular polysaccharide production, and carbohydrate metabolism (252 proteins enriched)	129 proteins	[44]

However, the molecular basis governing this selectivity remains largely elusive: what determines the preferential packaging of certain proteins into *S. mutans* MVs over others, and whether sorting signals or receptors akin to those in other Gram‑positive bacteria exist? Furthermore, most proteomic studies have been conducted under limited experimental conditions, lacking systematic multi‑factorial designs. Future investigations are required to distinguish general sorting principles from condition‑specific regulatory mechanisms.

### Lipids

Although the lipid bilayer framework of *S. mutans* MVs originates from the parental cytoplasmic membrane, vesicle lipid composition might be selectively remodelled and structurally distinct from the native bacterial membrane. Lipidomic profiling has revealed unique physicochemical properties in *S. mutans* MVs, characterised by a specific accumulation of cardiolipin and specific metabolites such as flavonoids [36]. Notably, the enrichment of monounsaturated long-chain fatty acids markedly increases membrane fluidity. This specialised lipid configuration is considered a critical adaptive strategy for *S. mutans* to tolerate acidic microenvironments, thereby enhancing bacterial aciduricity and ecological competitiveness within cariogenic biofilms [36]. The lipid composition of *S. mutans* MVs reveals a pattern of both conservation and selectivity. The overall lipid profile closely resembles that of the cytoplasmic membrane, yet specific lipids are selectively enriched or depleted. For instance, wild‑type *S. mutans* MVs are consistently enriched in cardiolipin, but this enrichment is absent in Δ*srtA* and Δ*sfp* mutants; in contrast, polyketides and flavonoids are uniformly enriched in all MV types, irrespective of genetic background. All lipids reported under different conditions are listed in Table 4.

**Table 4.** Upregulated, downregulated and missing lipids in *S. mutans* MVs under different conditions.

Condition	Upregulated lipids	Reduced lipids	Missing lipids	Reference
Lipids in *S. mutans Smu_opuB* MVs compared with *S. mutans* UA159 at pH 7.5	Acylcarnitine (AcCa), ceramide G2 glycans (CerG2GNAc1), monoglyceride (MG), phosphatidylcholine (PC)	Lysophosphatidylethanolamine (LPE), lysophosphatidylglycerol (LPG), phosphatidylethanolamine (PE), wax esters (WE)	/	[32]
Lipids in *S. mutans Smu_opuB* MVs compared with *S. mutans* UA159 at pH 5.5	Ceramide G2 glycans (CerG2GNAc1), glycosyltransferase 3 (GT3), monoglyceride (MG)	Lysophosphatidylethanolamine (LPE), PE	/	[32]
Lipids in *S. mutans* strain NG8 Δ*srtA* MVs compared with corresponding cytoplasmic membrane	Glycerolipids, polyketides (flavonoids), neutral sphingolipids, triacylglycerides	Glycerophospholipids, particularly cardiolipin	Prenol lipids	[36]
Lipids in *S. mutans serotype c* WT strain NG8 MVs compared with corresponding cytoplasmic membrane	Cardiolipins, polyketides (flavonoids)	/	Sterol lipids, prenol lipids, saccharolipids	[36]
Lipids in *S. mutans* strain NG8 Δ*sfp* MVs compared with corresponding cytoplasmic membrane	Sterol lipids, polyketides (flavonoids), steroids, secosteroids	Glycerophospholipids, glycerolipids	Sterol lipids, sphingolipids, fatty acids, conjugates	[36]

Note: ‘Missing lipids’ indicate lipids detected in MVs from the corresponding wild-type strain but not detected in MVs from the knockout strain under the same experimental condition.

The functional relevance of this selective sorting, however, remains largely elusive. Cardiolipin enrichment has been hypothetically linked to acid tolerance, but direct experimental proof is lacking. Even more enigmatic is the consistent presence of polyketides and flavonoids: whether they are actively recruited into *S. mutans* MVs or passively carried as metabolic by‑products, and what biological roles they might serve, remain entirely open questions that warrant future investigation. Moreover, emerging evidence suggests that gene deletions such as *srtA* or *sfp* do not simply alter the abundance of individual lipids but instead remodel the entire lipid sorting landscape. The mechanisms and pathways underlying this remodelling are currently unclear.

### Nucleic acids

In addition to proteins and lipids, *S. mutans* MVs serve as critical carriers for bacterial genetic materials, including eDNA and various RNA subtypes. MV-associated eDNA acts as a fundamental structural scaffold that co-localises with extracellular glucans, stabilises biofilm matrix architecture, and maintains the three-dimensional integrity of cariogenic biofilm [25]. Furthermore, *S. mutans* MVs encapsulate diverse RNA species, including mRNA, tRNA, and lncRNA [61]. Emerging evidence indicates that the abundance and composition of nucleic acids associated with *S. mutans* MVs vary according to environmental and genetic conditions. Under acidic conditions, tRNA-Arg, tRNA-Glu, 16S rRNA, and protein-coding RNAs were more abundant, whereas tRNA-Ser, tRNA-Val, and 5S rRNA showed reduced abundance compared with neutral conditions [58]. All nucleic acids reported under different conditions are listed in Table 5. In addition, eDNA levels increased during early exponential growth and were approximately 10-fold higher in culture supernatants from the *S. mutans* Δ*smu_833* than from the wild type [25,27]. These condition-dependent changes suggest selective regulation of MV-associated nucleic-acid abundance, as summarised in Table 5.

**Table 5.** Differentially abundant nucleic acids associated with *S. mutans* MVs under different conditions.

Condition	Nucleic acids with increased abundance	Nucleic acids with decreased abundance	Reference
eDNA in *S. mutans* UA159 and NG8 from early-exponential-phase cultures compared with overnight cultures	eDNA increased approximately 2.82-fold	/	[25]
Nucleic acids in *S. mutans* UA159 Δ*smu_833* MVs compared with WT MVs	eDNA in culture supernatants increased approximately 10-fold	/	[27]
Small RNAs in *S. mutans* KCTC 3065/ATCC 25175 EVs	tRNA-Met Oligo 1, tRNA-Ile, tRNA-Met Oligo 3, tRNA-Val, tRNA-Asn	/	[55]
RNA in *S. mutans* UA159 MVs at pH 5.2 compared with pH 7.2	tRNA-Arg, tRNA-Glu, 16S rRNA, protein-coding RNAs	tRNA-Ser, tRNA-Val, 5S rRNA	[58]

Recent studies have shown that tRNA species packaged within *S. mutans-*derived MVs can be internalised by host cells (such as oral epithelial cells), where they subsequently promote cellular proliferation and accelerate wound healing [55]. In addition, the detection of small RNAs, including microRNAs, in *S. mutans*-derived MVs further supports the role of *S. mutans* MVs as novel signalling vehicles for intercellular communication. These vesicular small RNAs potentially mediate sophisticated cross-kingdom regulatory networks between bacteria and the host, possibly via RNA interference-dependent mechanisms [28,61].

In conclusion, the cargo composition of *S. mutans* MVs is highly selective and subject to dynamic regulation. Proteins, lipids, and nucleic acids are not incorporated randomly but selectively sorted into *S. mutans* MVs (Figure 3), which may influence their physicochemical properties and biological activities. However, the mechanisms underlying cargo selection remain largely unclear. This is especially evident for RNA cargos: it remains unknown whether RNA packaging is mediated by sequence features, RNA secondary structures, or RNA-binding proteins. In addition, the increase in MV-associated mRNA under acidic conditions may arise from selective RNA loading or nonspecific changes during *S. mutans* MV production. Furthermore, although eDNA in *S. mutans* MVs has been relatively well characterised, the potential competition or synergy between RNA and eDNA during cargo sorting has not been explored.

**Figure 3.**
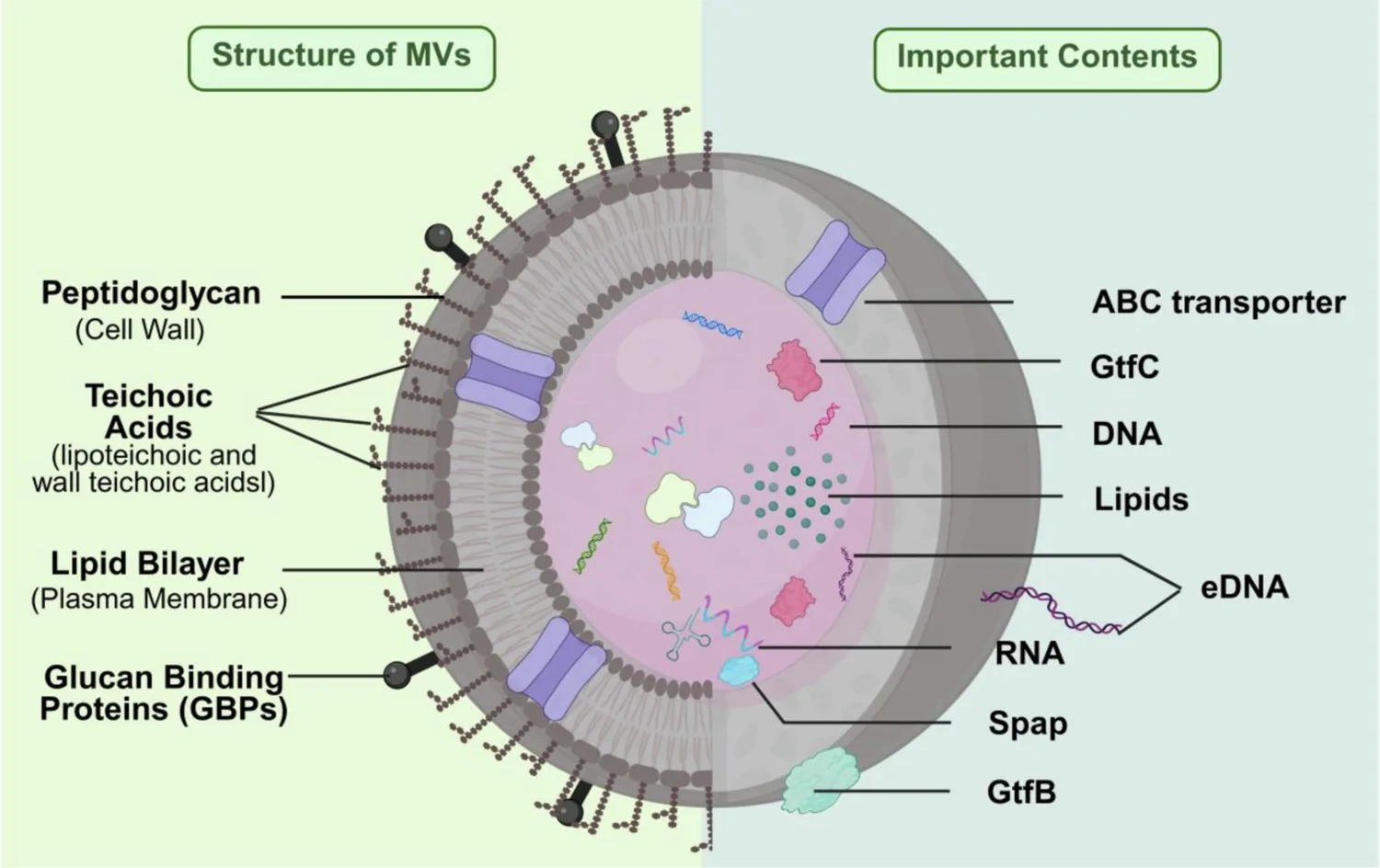
*S. mutans* MV Structure and Cargos. The figure shows that structures of *S. mutans* MVs contain peptidoglycans, teichoic acids, the lipid bilayer, and glucan binding proteins. Besides, MVs contain important cargos, such as ABC transporter, GtfB, GtfC, SpaP, lipids, DNA, eDNA and RNA. Created in BioRender.

As summarised in Table 6, the functional significance of *S. mutans* MV cargos remains incompletely defined. Gtfs and eDNA are the most directly linked to glucan synthesis, biofilm-matrix formation, and early colonisation by oral microbes. Many proteins, lipids, and nucleic acids identified by proteomic, lipidomic, and RNA-based analyses are associated with stress adaptation, adhesion, metabolism, or interspecies interactions, but their specific contributions to these effects have not been verified. Consequently, variation in MV cargo composition may contribute to the functional disparities reported across studies, but the mere presence of a cargo alone does not demonstrate its functional role. Future studies should integrate cargo profiling with targeted depletion, complementation, or reconstitution approaches to establish causal links between specific *S. mutans* MV cargos and biological outcomes.

**Table 6.** Major cargos and functional findings reported for *S. mutans* MVs.

Study	Major cargos	Major functional findings
Liao et al. [25]	eDNA, P1, GbpB, GbpC and GTFs	eDNA is a functional component within *S. mutans* biofilms, and that eDNA also plays a substantial role in bacterial adherence and biofilm initiation.
Senpuku et al. [46]	GTFs and complex DNA	The short-sized DNA associated with *S. mutans* MVs and GTFs are important contributors to the biofilm formation and may be additional targets for the prevention of oral biofilm-associated diseases.
Rainey et al. [27]	eDNA and glucan-associated components	SMU_833 regulates *S. mutans* biofilm matrix dynamics and virulence by modulating Gtf-dependent glucan production and MV-associated eDNA release.
Morales-Aparicio et al. [36]	proteins including GTFs, GbpD, Ftf, FruA, AtlA, SpaP; lipids including ardiolipin, fatty acids, and polyketide flavonoids	SrtA and Sfp altered MV production, size and cargo composition; Δ*srtA* MVs were larger and showed marked lipid remodelling.
Cao et al. [43]	pH-dependent proteins and metabolites, including virulence-associated proteins, metabolic enzymes, and ABC-transporter-related components	Acidic conditions increased MV yield, reduced size and altered 140 proteins and 37 metabolites*; S. mutans* MVs promoted EPS production and bacterial attachment.
Wu et al. [50]	GtfB and GtfC	*S. mutans* MVs enhance bovine dentin demineralisation provoked by *C. albicans* biofilms and enhance the protein and metabolite expression of *C. albicans* related to carbohydrate metabolism.
Nakamura et al. [49]	GtfB, GtfC and PAc	GtfC influenced MV size and biofilm-promoting activity; intranasal MVs induced Gtf- and PAc-reactive antibodies.
Wen et al. [48]	eDNA, GTFs and biofilm-associated proteins	sfp, bacA, bacA2, dac and pdeA modulated MV production; strain 27-3 showed a hypervesiculation phenotype.
Iwabuchi et al. [26]	GtfC, other GTFs and eDNA	Initial pH and the acid can adjust pH altered *S. mutans* MV size, Gtf abundance and biofilm-promoting activity.
Cui et al. [45]	GTFs implicated through Δ*gtfBC* MVs	*S. mutans* MVs inhibited the formation and reduced the biomass of *S. gordonii* and *S. sanguinis* biofilms, with the inhibitory effect involving MV-associated GTFs and eDNA.
Mizuta and Suzuki [53]	GtfB/GtfC and eDNA	*S. mutans* MVs containing insoluble glucan synthase and eDNA contribute to the promotion of initial attachment and colonisation of *Actinomyces oris.*
Leiva-Sabadini et al. [28]	LuxS, GTFs, and TrxB	Collagen-bound *S. mutans* biofilms produced smaller and less abundant MVs with altered protein cargo. These MVs were enriched in proteins associated with adhesion, glucan synthesis, stress tolerance, and quorum sensing. Collagen glycation further changed MV aggregation and cargo composition.
Nagasawa et al. [41]	Gtf-I/Gtf-SI and Com-induced secreted and cytoplasmic proteins	CSP/XIP signalling induced LytF-dependent autolysis and MV release from a bacterial subpopulation; *S. mutans* MVs differed functionally according to their induction route.
Wang et al. [32]	opuB- and pH-dependent proteins and lipids, including monoglycerides and acylcarnitines	OpuB transporter may enhance membrane stability under acidic conditions by regulating membrane lipid synthesis.
Iwabuchi et al. [54]	GTFs	Extracellular DNA, *S. mutans* MVs, and dead cells are factors that promote *S. aureus* biofilm formation under harsh conditions containing NaCl.
Oh et al [55]	RNA (especially tRNAs)	*S. mutans* MVs enhanced epithelial cell proliferation, migration, and wound healing in oral mucosal organoids and mouse skin.
Hashizume-Takizawa et al. [56]	GtfB, GtfC and other immunogenic proteins	Eggshell-derived hydroxyapatite enhanced MVs-specific mucosal IgA and systemic IgG responses; induced antibodies neutralised MVs-dependent biofilm formation.
Boone et al. [58]	tRNA, rRNA, ncRNA, tmRNA and protein-coding RNA	Acidic stress increased MV production and total MV-associated RNA and altered RNA-class distribution; individual tRNAs showed selective packaging.
Dufour et al. [47]	MVs were enriched in Pep299 and stress- and persistence-associated proteins.	CSP increased *S. mutans* MV production and remodelled their cargos. CSP-induced *S. mutans* MVs promoted persister formation and antibiotic survival in a dose-dependent manner. Comparison of Pep299-overexpressing and Δ Pep 299 MVs identifies Pep299 as a key determinant of this effect.
Cui et al. [44]	GtfC, GtfB, Ftf, MurD, and AtlA	Normoxic *S. mutans* MVs promote initial adhesion and early biofilm formation by enhancing glucan synthesis, bacterial self-aggregation, and surface adhesion.

## Biological functions

### *S. mutans* MVs influence biofilm formation

*S. mutans* MVs contribute profoundly to the construction and maturation of extracellular matrices of cariogenic biofilms. *S. mutans* MVs carry GtfB and encapsulate GtfC , and these two enzymes synergistically facilitate sucrose metabolism and insoluble glucan synthesis. This process remodels the physicochemical properties of the biofilm matrix and eventually enhances bacterial cariogenic virulence [53]. Similarly, a recent study revealed that normoxic stress promotes biofilm formation in *S. mutans* by enriching specific cargo, such as GtfC and SpaP, in its MVs [44]. These enriched cargos facilitate the bacterium’s initial colonisation and adhesion through both sucrose-dependent and sucrose-independent pathways. Notably, MVs can self-assemble with eDNA to form stable MV-eDNA complexes, which serve as structural scaffolds to mediate initial bacterial adhesion and aggregation, thereby promoting biofilm development. Functional assays have demonstrated that MV-eDNA complexes are capable of rescuing the defective biofilm phenotype of the *gtfBC*-deficient *S. mutans* UA159 strain, whereas eDNA depletion dramatically abolishes this compensatory effect [27]. Mechanistically, eDNA anchored on the *S. mutans* MV surface specifically recognises and binds matrix glucans, and such molecular colocalization substantially stabilises the three-dimensional architecture of cariogenic biofilm [27].

Emerging evidence has further identified autoinducer-2 (AI-2), a core quorum sensing signalling molecule, as a novel functional cargo of *S. mutans* MVs [62]. AI-2-containing MVs do not interfere with the growth of *S. mutans* but exert dose-dependent regulatory effects on biofilm formation. Specifically, high concentrations of AI-2-containing MVs significantly inhibit biofilm assembly [62]. Despite this observable phenotype, the precise molecular mechanisms underlying the AI-2-mediated inhibitory regulation remain poorly characterised and require further mechanistic exploration.

### *S. mutans* MVs participate in interspecies and cross-kingdom interactions

As pivotal signalling vesicles secreted by bacteria, *S. mutans* MVs exert multifaceted regulatory effects on the ecological succession of the oral microbiome by mediating complex interspecies competition and cooperation.

*S. mutans* MVs harvested in 16-hour standard cultures enable the pathogen to gain competitive advantage by suppressing biofilm formation and hydrogen peroxide production over commensal oral bacteria such as *Streptococcus sanguinis* [45]. New findings have further uncovered a context-dependent regulatory effect of *S. mutans* MVs on *Streptococcus sanguinis* adhesion: promoting bacterial adhesion during the planktonic phase while inhibiting it during biofilm maturation. This biphasic regulatory activity may serve as an adaptive ecological strategy that facilitates niche colonisation by *S. mutans* during the initial stages of cariogenesis [63]. In contrast, under environmental stress conditions such as high salinity, *S. mutans* MVs act synergistically with dead cells and eDNA to promote colonisation and survival of *Staphylococcus aureus* [54]. Additionally, *S. mutans* MVs directly facilitate the initial adhesion of *Actinomyces* species to biofilm surfaces via insoluble glucan synthase and eDNA in their cargo [53]. Collectively, these findings highlight the key role of *S. mutans* MVs as structural and biochemical ‘bridges’ that mediate the assembly of multispecies cariogenic biofilms [53].

In terms of cross-kingdom interactions, *S. mutans* MVs act as pivotal mediators in bacterial-fungal crosstalk. Wu et al. verified that *S. mutans* MVs promote biofilm development, acidogenicity, and hyphal morphogenesis of *Candida albicans* by modulating fungal amino acid and carbohydrate metabolism, ultimately accelerating the demineralisation of bovine dentin [51]. Mechanistically, using ubiquitin-based proteomic analysis, Zhang et al. revealed that *S. mutans* MVs optimise fungal energy metabolism by upregulating the ubiquitination of key glycolytic proteins and simultaneously inducing the degradation of superoxide dismutase 3 [57]. This triggers a moderate elevation of intracellular reactive oxygen species (ROS) in fungi, which further function as secondary signalling molecules to drive fungal proliferation and the formation of compact bacteria-fungal dual-species biofilms [57].

In the past few years, MVs-mediated horizontal gene transfer (HGT) has emerged as a research hotspot in microbiology. While MVs from diverse bacterial species have been validated as effective vectors for HGT that protect eDNA from degradation and facilitate the transmission of antibiotic resistance genes, the occurrence and underlying mechanism of HGT mediated by *S. mutans* MVs remain largely uncharacterised [64].

### Interactions between *S. mutans* MVs and the host

*S. mutans* MVs exert dichotomous regulatory effects in host-pathogen interactions, exhibiting both detrimental pathogenic properties and beneficial therapeutic potential. Pathogenically, *S. mutans* MVs possess potent pro-inflammatory, immune-evasive, cancer-promoting, and systemic tissue-damaging activities. Specifically, these MVs activate the NLRP3 and AIM2 inflammasomes in macrophages, triggering the secretion of IL-1β and reprogramming cellular energy metabolism towards enhanced glycolysis. Meanwhile, they suppress the phagocytic capacity of macrophages to promote bacterial colonisation [52]. Furthermore, *S. mutans* MVs dampen interferon expression via upregulation of the EGFR-ERK pathway, thereby exacerbating host susceptibility to HSV-1 infection [59]. A study by Cao et al. demonstrated that co-administration of *S. mutans* MVs accelerates the growth and migration of oral squamous cell carcinoma (OSCC) *in vivo* [65]. Findings from transcriptomic analysis, loss-of-function experiments and DIA proteomics indicated that this phenomenon might be closely related to *β*-catenin signalling [65]. These findings support that *S. mutans* MVs might promote OSCC progression through *β*-catenin-related pathway. Systemically, *S. mutans*-derived MVs can be effectively internalised by vascular endothelial cells. They disrupt the NO/ET-1 balance by inhibiting the Akt/eNOS pathway and activating the p38 MAPK cascade, resulting in impaired endothelium-dependent vasodilation. These findings provide direct molecular evidence supporting the mechanistic association between dental caries and cardiovascular diseases [30]. A recent study revealed that Pep299-containing *S. mutans* MVs, which can stimulate the enrichment of peptide Pep299 under CSP-induced cultures, play an important role in antibiotic persistence by quorum-regulation [47]. This finding provides a possibility for the future bacterial engineering to regulate the oral microecology and prevent antibiotic persistence by constructing gene-deletion strains like Δ299 *S. mutans*.

In contrast, *S. mutans* MVs show promising application prospects in immune regulation and tissue repair. Accumulating studies have demonstrated that GtfC-enriched *S. mutans* MVs act as potent mucosal immunogens. When combined with an eggshell-derived nano-hydroxyapatite adjuvant, they elicit robust mucosal IgA and IgG immune responses, highlighting the feasibility of developing MVs-based anti-caries vaccines [56]. Additionally, tRNA encapsulated within *S. mutans*-derived MVs promotes oral epithelial cell migration and accelerates cutaneous wound healing in mice through a TLR3-dependent signalling pathway, further expanding their potential applications in tissue engineering and regenerative medicine [55].

Collectively, *S. mutans* MVs exert both positive and negative effects on biological activities involving the microbiota and even the hosts (Figure 4). On one hand, MVs can promote *S. mutans* biofilm formation, enhance interspecies and cross-kingdom interactions, and contribute to host pathogenicity. On the other hand, MVs may have positive potentials in medical therapy and wound healing. However, further exploration of MV biological functions and the underlying pathways is still needed in the future. These apparently opposite effects may be related to differences in cargo, dose, and target cells. Further studies are needed to identify the factors that determine whether these vesicles exert pathogenic or beneficial effects and to clarify the underlying mechanisms.

**Figure 4.**
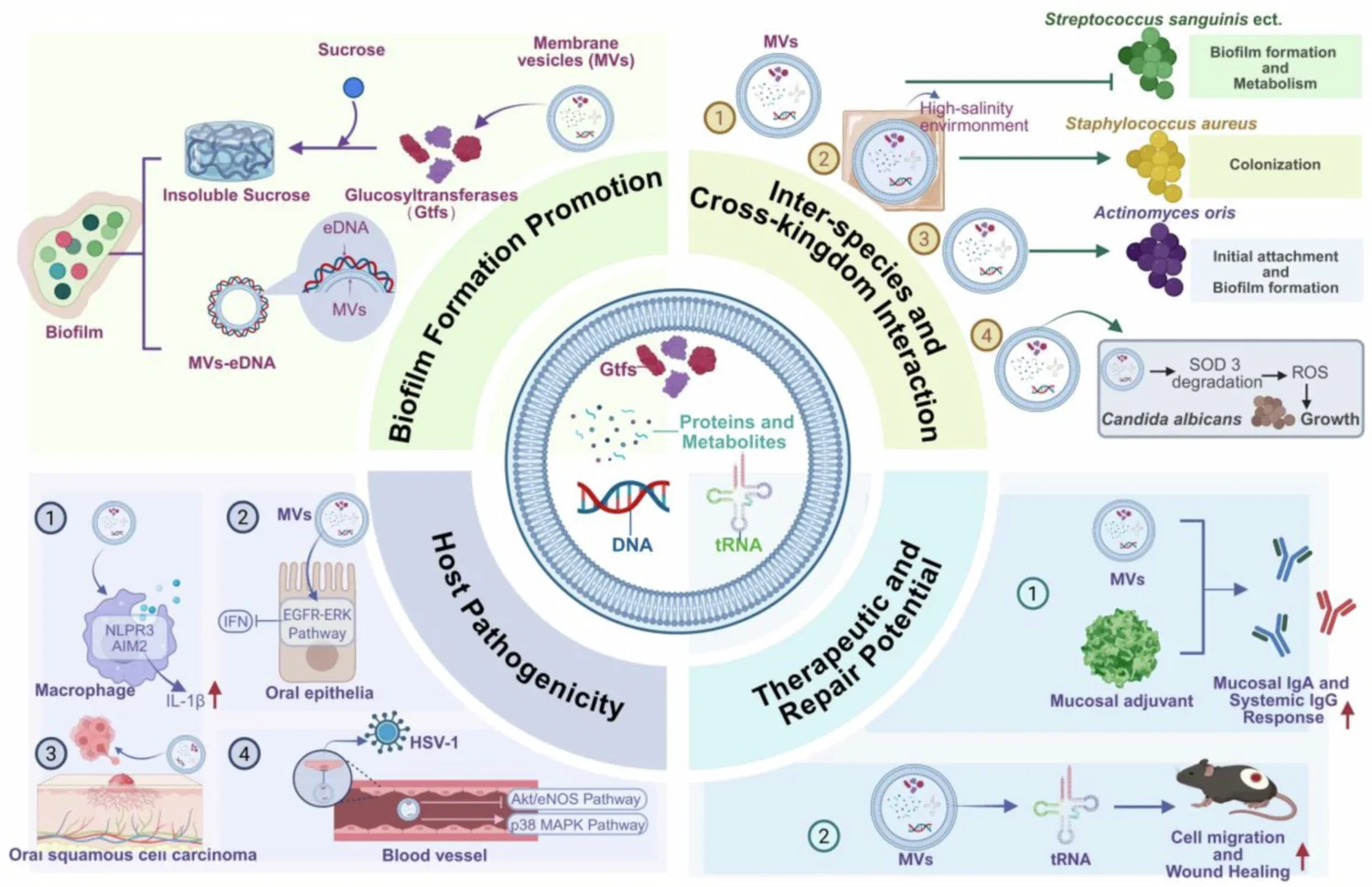
Biological Functions of *S. mutans* MVs. *S. mutans* MVs have been proved essential in biofilm formation promotion, interspecies and cross-kingdom interaction, inducing host pathogenicity, and potential in therapy or repairment. Created in BioRender.

## Challenges

As a novel signalling carrier, *S. mutans* MVs present numerous scientific questions that need to be addressed to further elucidate their pathological and physiological roles in oral microecology and systemic diseases.

(1) The mass production and stability enhancement of *S. mutans* MVs remain unachieved, which limits their application and development. Standardised procedures for MV isolation, purification, and characterisation are therefore needed.
(2) Most existing studies remain at the level of descriptive observations or rely on inferences drawn from characteristics of other Gram‑positive bacteria. The robust direct evidence and in-depth mechanisms underlying *S. mutans* MV biogenesis and cargo selection are still unclear.
(3) Research on the ecological functions of *S. mutans* MVs in complex microbial community microenvironments are inadequate. Most existing studies are based on the single‑species biofilm models, whereas the roles of *S. mutans* MVs in the complex oral microecology remain unknown.
(4) Studies on the initiation and progression of dental caries have been largely confined to *in vitro* models. Moreover, whether *S. mutans* MV composition systemically differs between high and low cariogenicity *S. mutans* strains remains unclear.
(5) The relationship between *S. mutans* MVs and systemic health, as well as their interaction with host cells is still unknown. Furthermore, how *S. mutans* MVs achieve transmembrane transport and delivery requires further investigation.

All these challenges above can critically influence the development of future strategies for the prevention and treatment of dental caries.

## Conclusion and future perspectives

In summary, acting as efficient carriers of toxins and other bioactive factors, *S. mutans* MVs mediate a core mechanism by which bacteria survive, compete, and induce disease in complex microenvironments. They not only regulate the bacterium’s own oral niches, but also deeply participate in complex interspecies communication, and microbe-host cross-kingdom interaction.

Although MVs increase the complexity of caries prevention and treatment, they also offer possibilities for new therapies. Future research should focus on standardised isolation of *S. mutans* MVs, mechanistic dissection, interaction networks, clinical translation, and interference strategies, in order to elucidate the precise molecular network underlying MV biogenesis and cargo sorting mechanisms, as well as to further unravel the molecular basis of bacteria‑bacteria/fungi and bacteria‑host interactions (particularly cross‑kingdom regulation) mediated by MVs. Advances in high-resolution imaging, single-vesicle technologies, multi-omics, and genetic perturbation provide opportunities to identify distinct *S. mutans* MV subpopulations and clarify their structure-function relationships. High-throughput vesicle omics approaches permit systematic dissection of MV protein, RNA, and lipid content, enabling the establishment of a cargo atlas for *S. mutans* MVs that underpins further mechanistic studies. Concurrently, single-vesicle flow cytometry and super-resolution imaging resolve MV heterogeneity at the individual level, allowing refined subpopulation typing. Looking forward, several emerging directions that remain underexplored may reshape the field. Leveraging digital and biological engineering to modify *S. mutans* MVs may contribute to achieving vesicles with tailored cargos, attenuated toxicity, and targeting specificity. A clearer mechanistic framework may support two complementary translational avenues: inhibiting the formation, release, cargo loading, or cellular uptake of pathogenic MVs could provide new strategies for controlling dental caries, whereas modified *S. mutans* MVs might be explored as cell-free vaccines or targeted delivery systems.

From a translational perspective, *S. mutans* MVs represent a highly promising class of nanomaterials which haven't been thoroughly explored. For successful translation, several key parameters, including reproducible production, cargo control, purity, safety, long-term stability, and targeting efficiency, must be carefully evaluated. Integrating mechanistic studies with standardised characterisation and clinically relevant models will be essential for the rational and safe application of *S. mutans* MVs.

## Data Availability

Data sharing is not applicable to this article as no new data were created or analysed in this study.
